# Safety and efficacy of glucagon-like peptide-1 receptor agonists among kidney transplant recipients: a systematic review and meta-analysis

**DOI:** 10.1093/ckj/sfae018

**Published:** 2024-02-02

**Authors:** Pajaree Krisanapan, Supawadee Suppadungsuk, Kanokporn Sanpawithayakul, Charat Thongprayoon, Pattharawin Pattharanitima, Supawit Tangpanithandee, Michael A Mao, Jing Miao, Wisit Cheungpasitporn

**Affiliations:** Division of Nephrology and Hypertension, Department of Medicine, Mayo Clinic, Rochester, MN, USA; Division of Nephrology, Department of Internal Medicine, Faculty of Medicine, Thammasat University, Pathum Thani, Thailand; Division of Nephrology, Department of Internal Medicine, Thammasat University Hospital, Pathum Thani, Thailand; Division of Nephrology and Hypertension, Department of Medicine, Mayo Clinic, Rochester, MN, USA; Chakri Naruebodindra Medical Institute, Faculty of Medicine Ramathibodi Hospital, Mahidol University, Samut Prakan Thailand; Division of Endocrinology and Metabolism, Department of Internal Medicine, Faculty of Medicine, Thammasat University, Pathum Thani, Thailand; Department of Clinical Epidemiology, Faculty of Medicine, Thammasat University, Pathum Thani, Thailand; Division of Nephrology and Hypertension, Department of Medicine, Mayo Clinic, Rochester, MN, USA; Division of Nephrology, Department of Internal Medicine, Faculty of Medicine, Thammasat University, Pathum Thani, Thailand; Division of Nephrology and Hypertension, Department of Medicine, Mayo Clinic, Rochester, MN, USA; Chakri Naruebodindra Medical Institute, Faculty of Medicine Ramathibodi Hospital, Mahidol University, Samut Prakan Thailand; Division of Nephrology and Hypertension, Department of Medicine, Mayo Clinic, Jacksonville, FL, USA; Division of Nephrology and Hypertension, Department of Medicine, Mayo Clinic, Rochester, MN, USA; Division of Nephrology and Hypertension, Department of Medicine, Mayo Clinic, Rochester, MN, USA

**Keywords:** GLP-1RAs, glucagon-like peptide-1 receptor agonist, kidney transplantation, Type 2 diabetes mellitus (T2DM), post-transplant diabetes mellitus (PTDM)

## Abstract

**Background:**

Evidence supporting glucagon-like peptide-1 receptor agonists (GLP-1RAs) in kidney transplant recipients (KTRs) remains scarce. This systematic review and meta-analysis aims to evaluate the safety and efficacy of GLP-1RAs in this population.

**Methods:**

A comprehensive literature search was conducted in the MEDLINE, Embase and Cochrane databases from inception through May 2023. Clinical trials and observational studies that reported on the safety or efficacy outcomes of GLP-1RAs in adult KTRs were included. Kidney graft function, glycaemic and metabolic parameters, weight, cardiovascular outcomes and adverse events were evaluated. Outcome measures used for analysis included pooled odds ratios (ORs) with 95% confidence intervals (CIs) for dichotomous outcomes and standardized mean difference (SMD) or mean difference (MD) with 95% CI for continuous outcomes. The protocol was registered in the International Prospective Register of Systematic Reviews (CRD 42023426190).

**Results:**

Nine cohort studies with a total of 338 KTRs were included. The median follow-up was 12 months (interquartile range 6–23). While treatment with GLP-1RAs did not yield a significant change in estimated glomerular filtration rate [SMD −0.07 ml/min/1.73 m^2^ (95% CI −0.64–0.50)] or creatinine [SMD −0.08 mg/dl (95% CI −0.44–0.28)], they were associated with a significant decrease in urine protein:creatinine ratio [SMD −0.47 (95% CI −0.77 to −0.18)] and haemoglobin A1c levels [MD −0.85% (95% CI −1.41 to −0.28)]. Total daily insulin dose, weight and body mass index also decreased significantly. Tacrolimus levels remained stable [MD −0.43 ng/ml (95% CI −0.99 to 0.13)]. Side effects were primarily nausea and vomiting (17.6%), diarrhoea (7.6%) and injection site pain (5.4%).

**Conclusions:**

GLP-1RAs are effective in reducing proteinuria, improving glycaemic control and supporting weight loss in KTRs, without altering tacrolimus levels. Gastrointestinal symptoms are the main side effects.

## Graphical Abstract

**Graphical Abstract:**
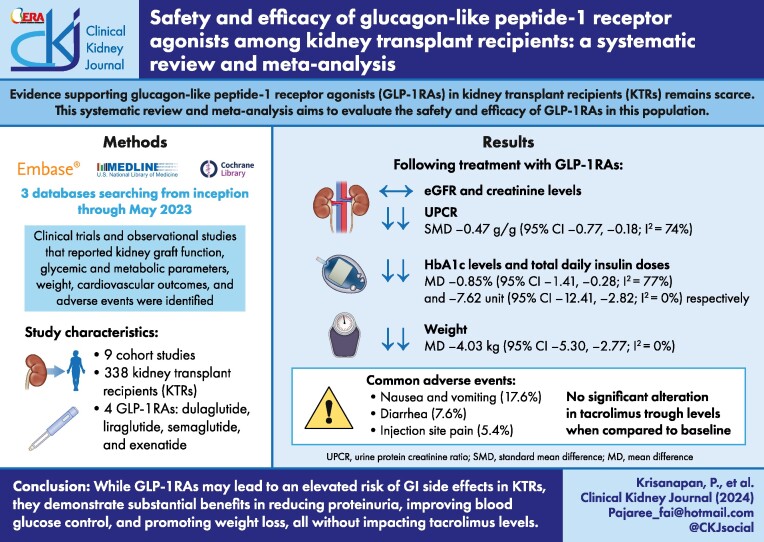

KEY LEARNING POINTS **What was known:**

- GLP-1RAs have been shown to be effective for glycaemic control and weight reduction in the general type 2 diabetes mellitus (T2DM) population.
- There is limited data on the safety and efficacy of GLP-1RAs in kidney transplant recipients (KTRs), a population at high risk for diabetes and its complications.
- Concerns also existed about the potential interaction of GLP-1RAs with immunosuppressive agents. A study was needed to evaluate their safety and efficacy in KTRs.

**This study adds:**

- GLP-1RAs are generally safe for use in KTRs. There are no significant alterations in immunosuppressive drug levels and their side-effect profile is similar to that of the general population.
- This study demonstrates that GLP-1RAs are effective in reducing proteinuria, improving glycaemic control and promoting weight loss in KTRs.
- No significant long-term cardiovascular or mortality outcome differences were observed. Further studies with extended follow-up are needed.

**Potential impact:**

- This study supports the inclusion of GLP-1RAs as a treatment option for T2DM in KTRs, thus expanding the therapeutic arsenal for this high-risk population.
- These findings may lead to protocol adjustments in the management of KTRs, particularly in the titration of immunosuppressive agents and GLP-1RAs.
- This study sets the stage for larger, controlled trials to confirm these findings and to explore long-term cardiovascular and mortality outcomes that would potentially impact future guidelines.

## INTRODUCTION

Kidney transplantation is currently widely acknowledged as the optimal kidney replacement therapy option. It offers superior survival outcomes, improved quality of life and cost-effectiveness when compared with maintenance dialysis for patients with end-stage kidney disease (ESKD) [1–4]. Diabetes mellitus (DM) is recognized as the leading cause of ESKD worldwide, contributing to 50–60% of global ESKD cases [5, 6]. In kidney transplant recipients (KTRs) with pre-existing type 2 diabetes mellitus (T2DM), this disease burden translates to the observed decreased survival rates compared with non-DM counterparts [7]. This disparity in survival outcomes can be chiefly attributed to the established correlation between DM and elevated cardiovascular (CV) risks. The increased CV risk associated with DM has been demonstrated to not only affect ESKD patients, but also lead to increased mortality rates across the general population [5, 8–10].

In KTRs, the necessary post-transplantation use of glucocorticoids and calcineurin inhibitors (CNIs) exacerbates the risk of hyperglycaemia and new-onset diabetes. Glucocorticoids have been demonstrated to induce hyperglycaemia by exacerbating insulin resistance, impairing α-cell and β-cell function and inhibiting the incretin effect [10–13]. CNIs, particularly tacrolimus, appear to have an adverse effect on β-cell function that results in reduced insulin secretion [11, 14]. Consequently, post-transplant diabetes mellitus (PTDM) has become increasingly common after kidney transplantation, and it can lead to diabetic kidney disease and allograft dysfunction [3, 15–17]. The estimated prevalence of pretransplant DM and PTDM ranges from ≈10 to 30% of KTRs, and this has become an increasingly significant barrier to improving post-transplant outcomes due to their association with increased CV risk, mortality and healthcare burden [3, 16].

Glucagon-like peptide-1 (GLP-1) is a natural incretin hormone secreted by the neuroendocrine L-cells located in the distal intestine following a meal [18, 19]. GLP-1 receptor agonists (GLP-1RAs) not only stimulate insulin release through a glucose-dependent mechanism and suppress glucagon release, but also induce weight loss by slowing gastric emptying time and suppressing appetite [20–22]. GLP-1RAs have demonstrated effectiveness in improving glycaemic control, reducing major adverse cardiac events (MACE), lowering all-cause mortality, minimizing hospitalizations due to heart failure and slowing the progression of renal dysfunction in high-CV-risk individuals with T2DM [22–27]. Furthermore, there is strong evidence that GLP-1RAs protect β-cells from glucocorticoid-induced injury and *in vitro* toxicity from CNIs [28]. Since GLP-1RAs can slow gastric emptying and thus potentially alter the absorption and efficacy of concurrent orally administered medications (such as tacrolimus, an antirejection drug with a tightly controlled therapeutic range), caution has been advised in their post-transplant use [12, 29].

To date, there is limited evidence regarding the safety and efficacy of GLP-1RAs in KTRs, primarily due to their general exclusion from randomized controlled trials (RCTs) [23–27, 30–33]. Additional treatment options for diabetes in KTRs are sorely needed to further improve post-transplant outcomes. Emerging studies suggest that GLP-1RAs may be safe and effective for managing glycaemic control and promoting weight loss, without significantly impacting immunosuppressive dosages in either T2DM and PTDM solid organ transplant recipients, particularly KTRs [34, 35]. Thus we conducted a systematic review and meta-analysis to comprehensively evaluate the safety and efficacy of GLP-1RAs in KTRs.

## MATERIALS AND METHODS

### Search strategy and study eligibility

The protocol for this systematic review and meta-analysis was registered with the International Prospective Register of Systematic Reviews (CRD42023426190). A pair of investigators (P.K. and S.S.) independently conducted a comprehensive search from inception through May 2023 utilizing the Ovid MEDLINE, Embase and Cochrane databases. In order to assess the safety and efficacy of GLP-1RAs in KTRs, the search terms included ‘GLP-1RAs OR liraglutide OR semaglutide OR dulaglutide OR lixisenatide OR exenatide OR albiglutide OR efpeglenatide’ AND ‘kidney transplant OR renal transplant’. The comprehensive search method is provided in Supplementary Table S1. The scope of the search was confined to human subjects without language limitations. Furthermore, a manual search into the references of the included studies and a meticulous search through relevant conference abstracts were undertaken to identify additional pertinent studies. The Preferred Reporting Items for Systematic Reviews and Meta-Analyses (PRISMA) statement was followed for the reporting of this systematic review [36].

This systematic review incorporated clinical trials and observational studies that evaluated the safety or efficacy outcomes of GLP-1RAs after kidney transplantation in adults ≥18 years of age. Primary outcomes included the efficacy of GLP-1RAs on mortality and CV diseases (e.g. myocardial infarction, stroke and heart failure), on kidney graft function [e.g. changes in creatinine, estimated glomerular filtration rate (eGFR), urine protein:creatinine ratio (UPCR) or 24-hour urine protein excretion], on glycaemic and metabolic outcomes (e.g. change in blood glucose or haemoglobin A1c (HbA1c), blood pressure (BP) and lipid profile] and on weight reduction. Secondary outcomes included tacrolimus levels, allograft rejection and any adverse events.

Excluded from this systematic review were case reports, editorials, reviews without original data and studies that primarily reported on recipients of other organ transplants that lacked a subgroup analysis specifically for KTRs. Eligibility assessment for retrieved studies was performed independently by the two investigators (P.K. and S.S.). Any disparities were resolved through collaborative discussion among all authors.

### Data extraction and quality assessment

A standardized data collection template was employed to extract the following variables from each study included in this analysis: study title; author(s); publication year; study design; country where the study was conducted; number of participants; duration of follow-up; name and dosage of GLP-1RAs; identity of the comparator drugs in the control group [including the type and dosage of insulin and/or other oral antihyperglycaemic drugs such as metformin, sulfonylurea, sodium–glucose co-transporter 2 (SGLT2) inhibitors and pioglitazone]; baseline characteristics including age, body weight, body mass index (BMI), laboratory test results, existing comorbidities and the type and dosage of immunosuppressive agents; and treatment outcomes involving GLP-1RAs and control groups, including changes in creatinine, eGFR, UPCR or 24-hour urine protein excretion, blood glucose or HbA1c, BP, body weight or BMI, tacrolimus levels, incidence of CV events, all-cause mortality and any adverse events (including allograft rejection).

For randomized controlled trials, the Cochrane Risk of Bias Tool [37] was utilized. For non-randomized studies, the Risk Of Bias In Non-randomized Studies of Interventions (ROBINS-I) tool [38] and Newcastle–Ottawa Scale [39] were utilized, as shown in Supplementary Tables S2 and S3. Funnel plots and Egger's test were used to examine for potential publication bias.

### Statistical analysis

The meta-analysis was performed with Comprehensive Meta-analysis version 3.3.070 (Biostat, Englewood, NJ, USA). For dichotomous outcomes, the study utilized odds ratios (ORs) with 95% confidence intervals (CIs) to express differences in effects. For continuous outcomes, the summary statistics for each outcome consisted of the mean change from baseline with associated standard deviations (SDs). Calculating the mean change within each group involved subtracting the final mean from the baseline mean. Mean differences (MDs) were employed when all studies reported the same continuous outcome using the same unit of measure. In other instances, standardized mean differences (SMDs) with accompanying 95% CIs were utilized. For the computation of the SD of mean change, we assumed a conservative correlation coefficient of 0.5, as suggested in the literature. Effect sizes were interpreted as follows: 0.2 indicated a small effect, 0.5 a moderate effect and 0.8 a large effect [40]. In cases where the original articles did not provide sufficient data, we sent requests to the investigators for additional data or calculated estimates from the available figures.

Heterogeneity was examined through the χ^2^ test and/or the *I*^2^ statistic. A value of *I*^2^ >50% or a *P*-value <.1 was indicative of significant heterogeneity. If the test for heterogeneity yielded significant results, the subsequent meta-analysis was conducted using a random effects model [41]. The possibility of publication bias was evaluated through a funnel plot analysis and Egger's test [42]. Subgroup analyses were performed for short-term (<12 months) and long-term (>12 months) outcomes. For all analyses, a *P*-value <.05 was considered statistically significant.

## RESULTS

### Study characteristics

**Figure 1:**
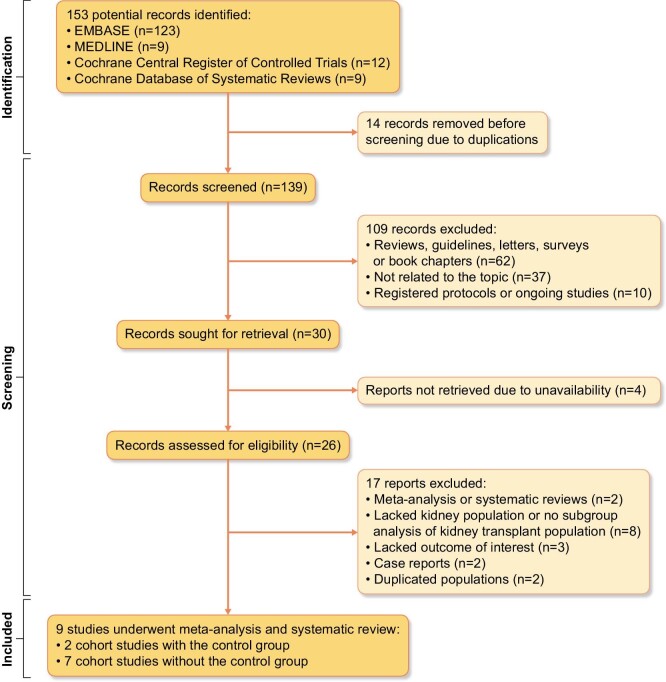
PRISMA flow of search methodology and selection process.

Our search strategy (Fig. 1) yielded a total of 153 potential articles, with 14 identified as duplicates. Therefore, 139 articles underwent screening based on their titles and abstracts. This resulted in the exclusion of 113 articles due to publication type, lack of topic relevance, ongoing studies or unavailability of the article. As a result, a total of 26 studies were included for full-length review. Of these, 17 studies were subsequently removed from the analysis for the following reasons: being a systematic review, meta-analysis or case report; lacking KTRs or subgroup analysis primarily focusing on KTRs; missing outcomes of interest; and duplication of the population. Thus this systematic review ultimately included nine studies with a sample size of 338 participants, including seven retrospective cohort studies without control groups [12, 43–48] and two retrospective cohort studies with control groups [49, 50]. The median follow-up time was 12 months [interquartile range (IQR) 6–23] with a range from 1 to 49.4 months as shown in Table 1.

**Table 1:** Characteristics of the included studies

Author (year)	Pinelli *et al*. (2013) [12]	Liou *et al*. (2018) [45]	Kukla *et al*. (2020) [44]	Gonzalez *et al*. (2021) [48]	Kim *et al*. (2021) [43]	Vigara *et al*. (2022)[47]	Mallik *et al*. (2023) [46]	Sato *et al*. (2023) [50]	Campana *et al*. (2023) [49]
Study type	Retrospective cohort	Retrospective cohort	Retrospective cohort	Retrospective cohort	Retrospective cohort	Retrospective cohort	Retrospective cohort	Retrospective cohort with a control group	Retrospective cohort with a control group
Country	USA	Taiwan	USA	USA	South Korea	Spain	UK	Japan	USA
Follow-up duration (months)	1	19.4 ± 7.6	12	12	6	12	26.5 ± 16.5	49.4	6
Participants, *n*	5 received GLP-1RAs	7 received GLP-1RAs	17 received GLP-1RAs	15 in total: 13 received GLP-1RAs, 2 received SGLT2 inhibitors	37 received GLP-1RAs	40 received GLP-1RAs^a^	23 received GLP-1RAs^b^	146 in total: 73 received GLP-1RAs, 73 controls (non-GLP-1RAs)	50 in total: 25 received GLP-1RAs, 25 controls (OADs)
GLP-1RAs	Liraglutide	Liraglutide	Liraglutide (82%), dulaglutide (12%), exenatide (6%)	Semaglutide (54%), liraglutide (31%), dulaglutide (15%)	Dulaglutide	Semaglutide (48%), liraglutide (32%), dulaglutide (20%)	Dulaglutide (74%), liraglutide (26%)	N/A	N/A
Doses of GLP-1RAs	Starting at 0.6 mg/day then optimized to 1.8 mg/day	Starting at 0.6 mg/day then optimized to 1.8 mg/day (mean dose 1.3 ± 0.5 mg/day)	Liraglutide 0.6–1.8 mg/day, dulaglutide 0.5–0.75 mg/week, exenatide 2 mg/week	N/A	0.75 mg/week (46%) and 1.5 mg/week (54%)	72.5% reached the maximum recommended dose of the drug	N/A	N/A	N/A
Type of DM	PTDM	N/A	65% PTDM, 18% T2DM	40% PTDM, 33% non-DM, 27% T2DM	T2DM	60% T2DM, 40% PTDM	N/A	T2DM	62% T2DM, 38% PTDM
Male, *n* (%)	3 (60)	N/A	14 (82.4)	10 (66.7)	18 (48.6)	21 (52.5)	15 (65.2)	102 (69.9)	32 (64)
Age (years), mean ± SD	55.4 ± 8.2	N/A	51.8 ± 16.1	54 ± 8.9	54.8 ± 8.5	62.8	56.5	57.4 ± 10.3	56.8
Weight (kg), mean ± SD	88.6 ± 17.0	78.0 ± 7.8	106.6 ± 19.8	92.4 ± 11.2	72.1 ± 11.6	93.0 ± 17.6	N/A	N/A	93.5 ± 17.3
BMI (kg/m^2^), mean ± SD	30.1 ± 6.2	29.4 ± 2.8	34.1 ± 3.9	31.2 ± 4.3	25.7 ± 3.4	35.8 ± 5.4	N/A	25.1 ± 3.5	N/A
Creatinine (mg/dl), mean ± SD	1.0 ± 0.2	1.2 ± 0.4	1.4 ± 0.4	1.3 ± 0.5	1.1 ± 0.3	N/A	N/A	N/A	N/A
eGFR (ml/min/1.73 m^2^), mean ± SD	87.6 ± 19.8	67.7 ± 18.7	53.0 ± 14.7	N/A	71.7 ± 18.5	46.1 ± 15.2	N/A	44.4 ± 13.6	N/A
UPCR (g/g), mean ± SD	N/A	1.38 ± 1.67	0.15 ± 0.06^c^	0.23 ± 0.39^c^	N/A	N/A	N/A	0.10 ± 0.70	N/A
Blood sugar (mg/dl), mean ± SD	91 ± 22	229 ± 39	139 ± 62	128 ± 49	145 ± 43	N/A	N/A	N/A	N/A
HbA1c (%), mean ± SD	N/A	10.0 ± 1.6	7.7 ± 1.1	6.7 ± 1.8	7.0 ± 0.9	7.7 ± 1.0	N/A	7.1 ± 1.1	N/A
Diabetic retinopathy, *n* (%)	N/A	N/A	N/A	N/A	29 (78)	N/A	10 (43)	N/A	N/A
Concomitant insulin use, *n* (%)	N/A	2 (29)	11 (65)	N/A	37 (100)	N/A	N/A	N/A	50 (100)
Total daily insulin dose (units), mean ± SD	N/A	N/A	63 ± 52	N/A	25 ± 12	49 ± 30	N/A	N/A	48 ± 32
OADs, *n* (%) Metformin Sulfonylurea SGLT2 inhibitors Pioglitazone	N/A	N/A	8 (47) 2 (12) 0 1 (6)	N/A	34 (92) 19 (51) 0 N/A	5 (13) 1 (3) 3 (8) N/A	N/A	N/A	N/A
Immunosuppressive drug use, *n* (%) Tacrolimus Cyclosporin Everolimus Mycophenolate Corticosteroids	5 (100) N/A N/A N/A 4 (80)	7 (100) N/A N/A N/A N/A	16 (94) 0 1 (6) 15 (88) 11 (65)	13 (100) N/A N/A N/A N/A	N/A	40 (100) N/A 1 (3) 38 (95) 38 (95)	N/A	CNIs 146 (100)^d^ 146 (100)^e^ 146 (100)	N/A
Source of funding	Research award program^f^	Taichung Veterans General Hospital	None	N/A	None	None	None	None	N/A

N/A, no data available; OADs, oral antidiabetic drugs.

^a^40 received GLP-1RAs at 6 months then decreased to 26 at 12 months; 50 included in safety analysis.

^b^23 received GLP-1RAs at 6 months then decreased to 19 at 12 months and 12 at 24 months.

^c^24-hour urine protein excretion (g/day).

^d^CNIs were defined as tacrolimus and cyclosporin.

^e^A combination of mycophenolate and everolimus.

^f^2011–2012 Eugene Applebaum College of Pharmacy & Health Sciences Faculty.

Among the 338 participants included in this systematic review, 240 individuals received GLP-1RAs and 98 individuals from two studies [49, 50] received non-GLP-1RAs. Almost all KTRs (98.5%) had DM, with 80% having pre-existing T2DM and 18.7% experiencing PTDM. Notably, only 5 of 338 participants from Gonzalez *et al*. [48] had no DM.

Overall, 65% of participants were male with a mean age of 57.0 ± 10.5 years (from 331 participants across eight studies [12, 43, 44, 46–50]). The mean body weight was 89.1 ± 18.8 kg (from 169 participants across seven studies [12, 43–45, 47–49]) and the mean BMI was 28.9 ± 6.0 kg/m^2^ (from 265 participants across seven studies [12, 43–45, 47, 48, 50]). In terms of baseline blood glucose control, the baseline HbA1c was 7.3 ± 1.2% (from 260 participants across six studies [43–45, 47, 48, 50]) and fasting blood glucose was 145.1 ± 55.0 mg/dl (from 79 participants across five studies [12, 43–45, 48]). It should be noted that only two of the included studies reported pre-existing diabetic retinopathy, affecting 65% of the 60 patients [43, 46].

Regarding baseline kidney function, the mean serum creatinine was 1.2 ± 0.4 mg/dl (from 79 participants across five studies [12, 43–45, 48]), the eGFR was 50.8 ± 18.7 ml/min/1.73 m^2^ (from 252 participants across six studies [12, 43–45, 47, 50]) and the UPCR was 0.16 ± 0.74 g/g (from 100 participants across five studies [44–48]). Detailed information regarding immunosuppressive agents was available in six studies with 228 participants [12, 44, 45, 47, 48, 50]. Tacrolimus was prescribed in 99% and corticosteroids in 85% of patients. Notably, only one study included a minority of combined kidney–heart transplant recipients (2/17) and kidney–liver recipients (1/17) [44].

Of the 240 patients who were prescribed GLP-1RAs, information about the specific medication administered was available for only 142 individuals across seven studies [12, 43–48]. Dulaglutide was the most commonly prescribed GLP-1RA (46.5%), with a weekly dosage of 0.75–1.5 mg [43, 44, 46–48]. Liraglutide (34.5%) was the second most frequently prescribed GLP-1RA, with doses ranging from 0.6 to 1.8 mg/day [12, 44–48], followed by semaglutide (18.3%) [47, 48] and exenatide (0.7%) [44]. The timing for initiation of GLP-1RAs after kidney transplantation was reported in only two studies as a mean of 7.7 ± 5.3 months [49] and a median of 24 months (IQR 15–61) [47].

### Efficacy of GLP-1RAs on kidney graft function

**Figure 2:**
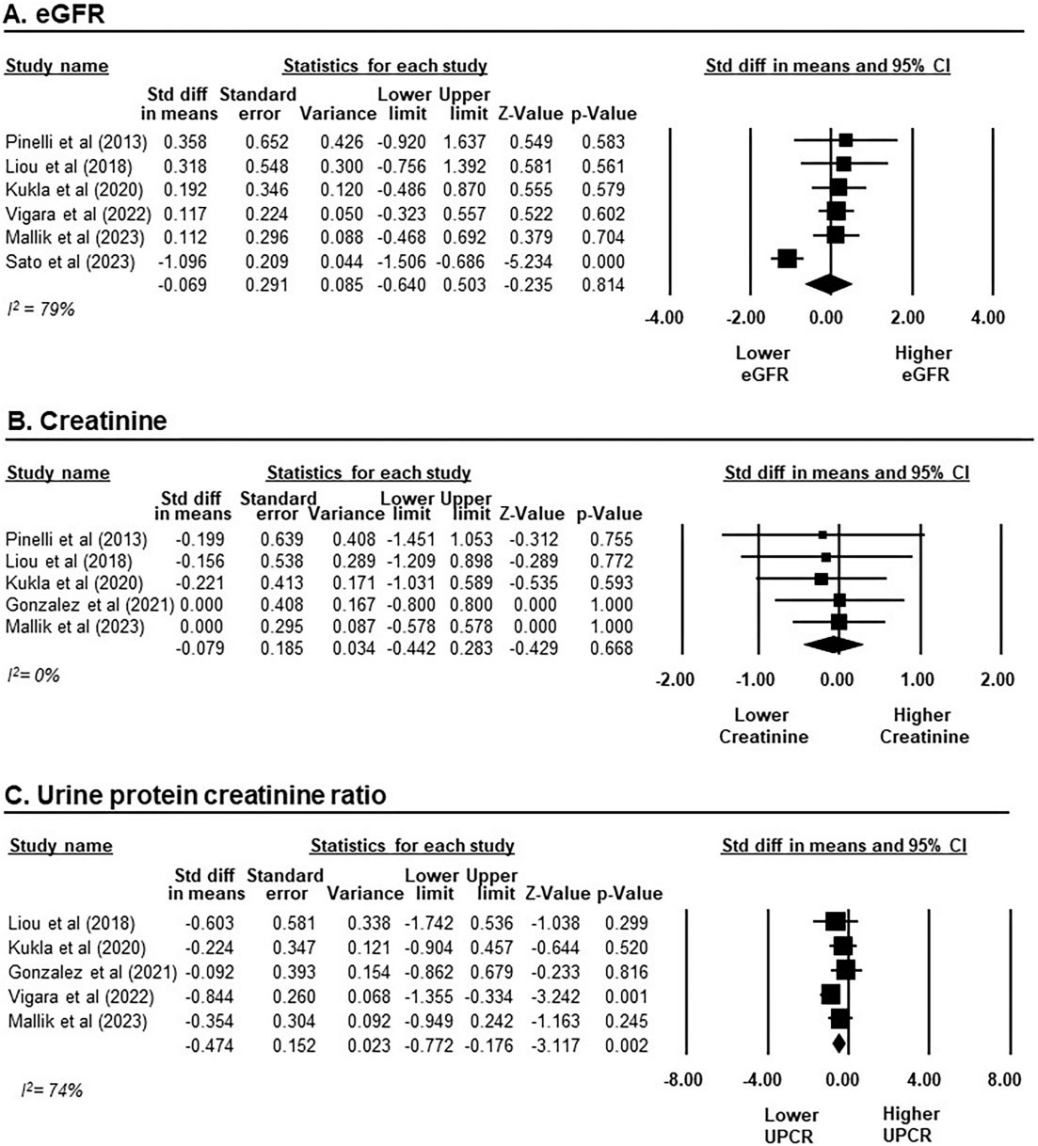
Changes in kidney graft function from baseline after GLP-1RAs treatment in KTRs. **(A)** eGFR presented on a scale ranging from −4 to 4 ml/min/1.73 m^2^. **(B)** Creatinine presented on a scale ranging from −2 to 2 mg/dl. **(C)** UPCR presented on a scale ranging from −8 to 8 g/g. Studies are identified by the name of the first author and the year of publication. SMDs were determined using the random effects model.

Six studies with a total of 165 individuals receiving GLP-1RAs were included for the meta-analysis on kidney graft function [12, 44–47, 50]. Overall, the change in eGFR after GLP-1RA treatment was comparable to baseline, with an SMD of −0.07 ml/min/1.73 m^2^ (95% CI −0.64–0.50), *P* = .814, *I*^2^ = 79%; Fig. 2A). A similar pattern was observed in the creatinine analysis, involving five studies with 65 participants [12, 44–46, 47]. The change in creatinine levels after GLP-1RA treatment was similar to that of baseline values, with an SMD of −0.08 mg/dl (95% CI −0.44–0.28, *P* = .668, *I*^2^ = 0%; Fig. 2B). However, GLP-1RA treatment did show a significant reduction in UPCR from baseline, with an SMD of −0.47 g/g (95% CI −0.77 to −0.18, *P* = .002, *I*^2^ = 74%; Fig. 2C) across five studies involving 100 participants [44–48].

Subgroup analyses by treatment duration (>12 or <12 months) were performed for both eGFR and creatinine. These analyses did not identify any statistically significant change in either eGFR or creatinine levels when compared with baseline, as shown in Supplementary Figs. S1 and S2.

### Efficacy of GLP-1RAs in glycaemic and metabolic outcomes

**Figure 3:**
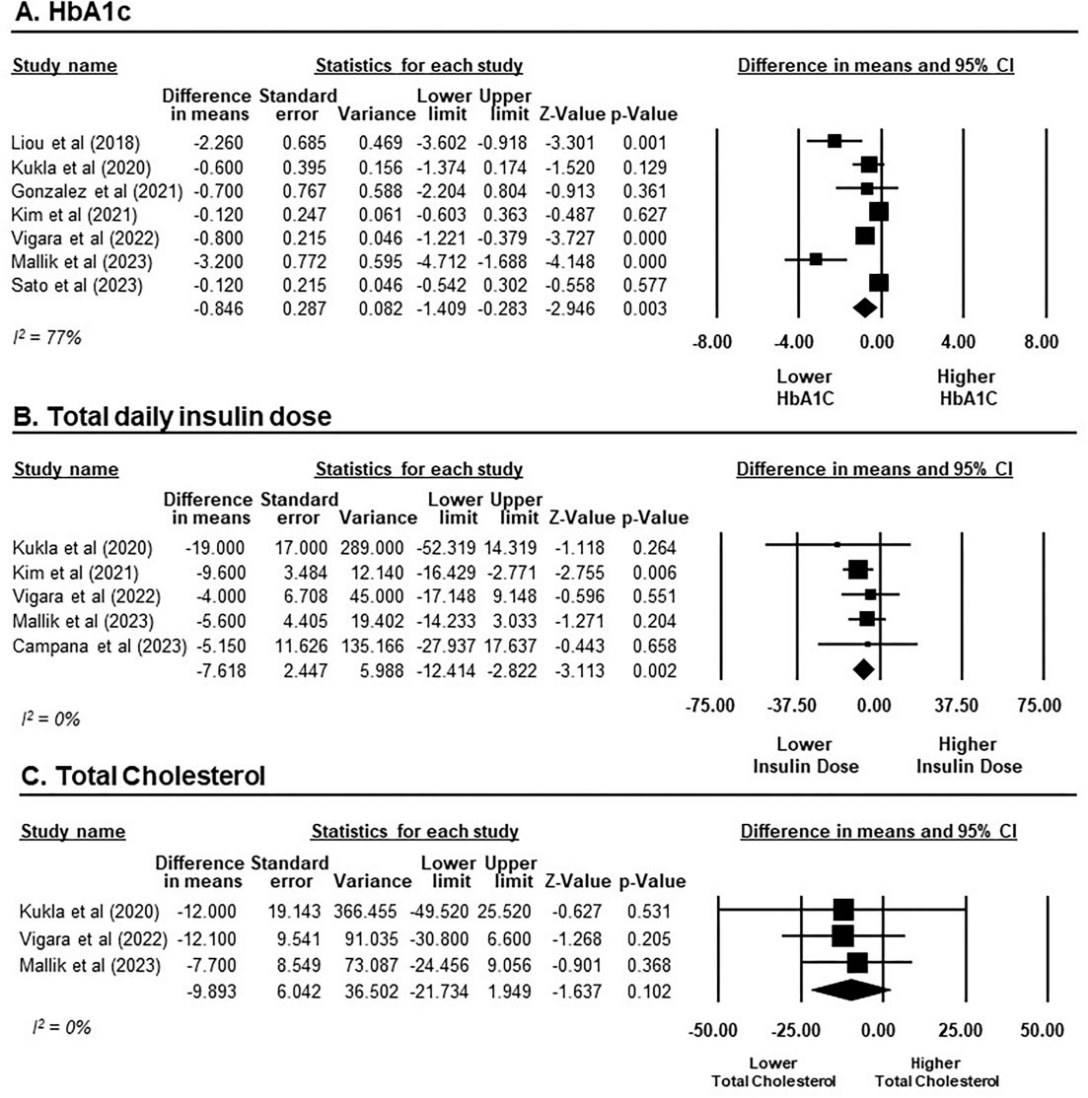
Changes in glycaemic and metabolic outcomes from baseline after GLP-1RAs treatment in KTRs. **(A)** HbA1c presented on a scale ranging from −8 to 8%. **(B)** Total daily insulin dose presented on a scale ranging from −75 to 75 units. **(C)** Total cholesterol presented on a scale ranging from −50 to 50 mg/dl. Studies are identified by the name of the first author and the year of publication. MDs were determined using the random effects model.

A total of 210 participants receiving GLP-1RAs from seven studies were included in the meta-analysis on HbA1c [43–48, 50]. Overall, there was a significant HbA1c reduction after treatment with GLP-1RAs, with an MD of −0.85% (95% CI −1.41 to −0.28, *P* = .003, *I*^2^ = 77%; Fig. 3A). In subgroup analysis, it is notable that GLP-1RAs achieved a statistically significant reduction in HbA1c levels only in the context of short-term treatment (<12 months), with an MD of −0.74% (95% CI −1.22 to −0.25, *P* = .003, *I*^2^ = 68%; six studies [43, 44, 46–48, 50]). In contrast, our findings did not reveal a statistically significant reduction in HbA1c during long-term treatment (>12 months), with an MD of −0.51% (95% CI −1.45–0.43, *P* = .287, *I*^2^ = 70%; two studies [46, 50]; Supplementary Fig. S3).

Regarding total daily insulin doses, our meta-analysis incorporated data from five studies comprising 138 participants receiving GLP-1RAs [43, 44, 46, 47, 49]. GLP-1RAs demonstrated a statistically significant reduction in total daily insulin dose from baseline, yielding an MD of −7.62 units (95% CI −12.41 to −2.82, *P* = .002, *I*^2^ = 0%) when administered as short-term treatment (Fig. 3B). Only one study provided data for long-term outcomes of GLP-1RAs on insulin use at 24 months; this revealed a reduction in the total daily insulin dose by −2.5 ± 23.7 units [46].

In terms of cholesterol profile, three studies involving 80 participants receiving GLP-1RAs reported short-term outcomes on total cholesterol [44, 46, 47]. GLP-1RAs did not exhibit a significant reduction in total cholesterol levels compared with baseline, with an MD of −9.89 mg/dl (95% CI −21.73–1.95, *P* = .102, *I*^2^ = 0%; Fig. 3C).

**Figure 4:**
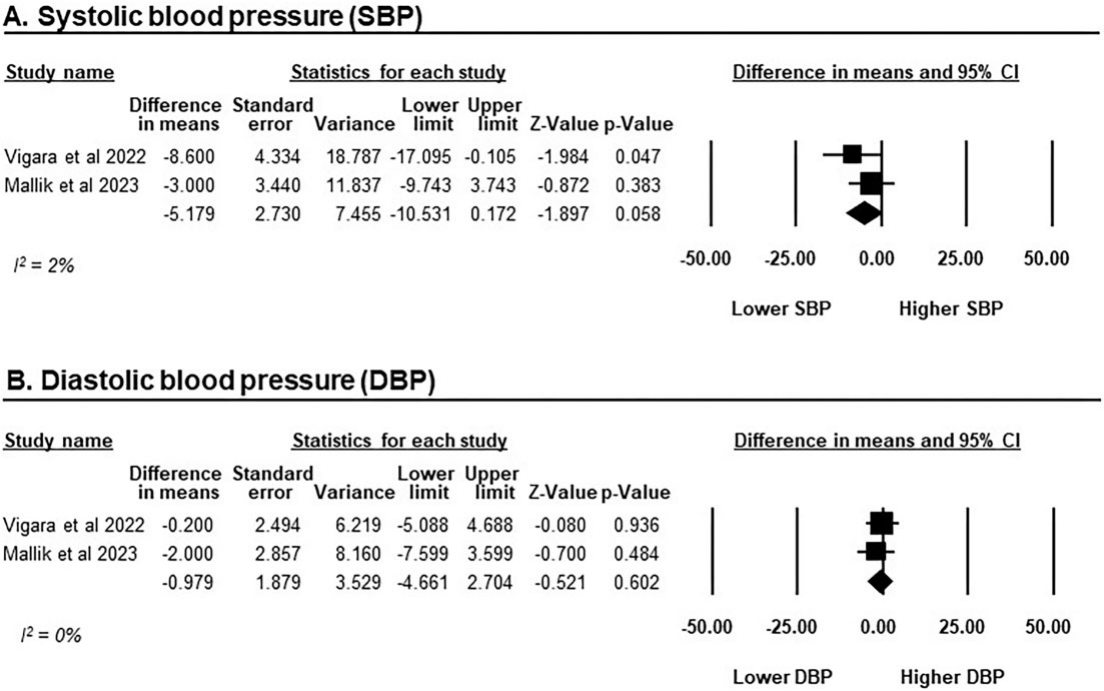
The changes in BP from baseline after GLP-1RAs treatment in KTRs. **(A)** SBP and **(B)** DBP. Studies are identified by the name of the first author and the year of publication and the outcomes are presented on a scale ranging from −50 to 50 mmHg. MDs were determined using the random effects model.

BP data were available in only two of the included studies, encompassing a total of 63 patients receiving GLP-1RAs [46, 47]. Following a 6-month course of treatment, GLP-1RAs did not reach a statistically significant decrease in systolic BP (SBP), with an MD of −5.12 mmHg (95% CI −10.53–0.17, *P* = .058, *I*^2^ = 2%). Similarly, there was no statistically significant impact on diastolic BP (DBP), with an MD of −0.98 mmHg (95% CI −4.66–2.70, *P* = .602, *I*^2^ = 0%; Fig. 4).

### Efficacy of GLP-1RAs in weight reduction

**Figure 5:**
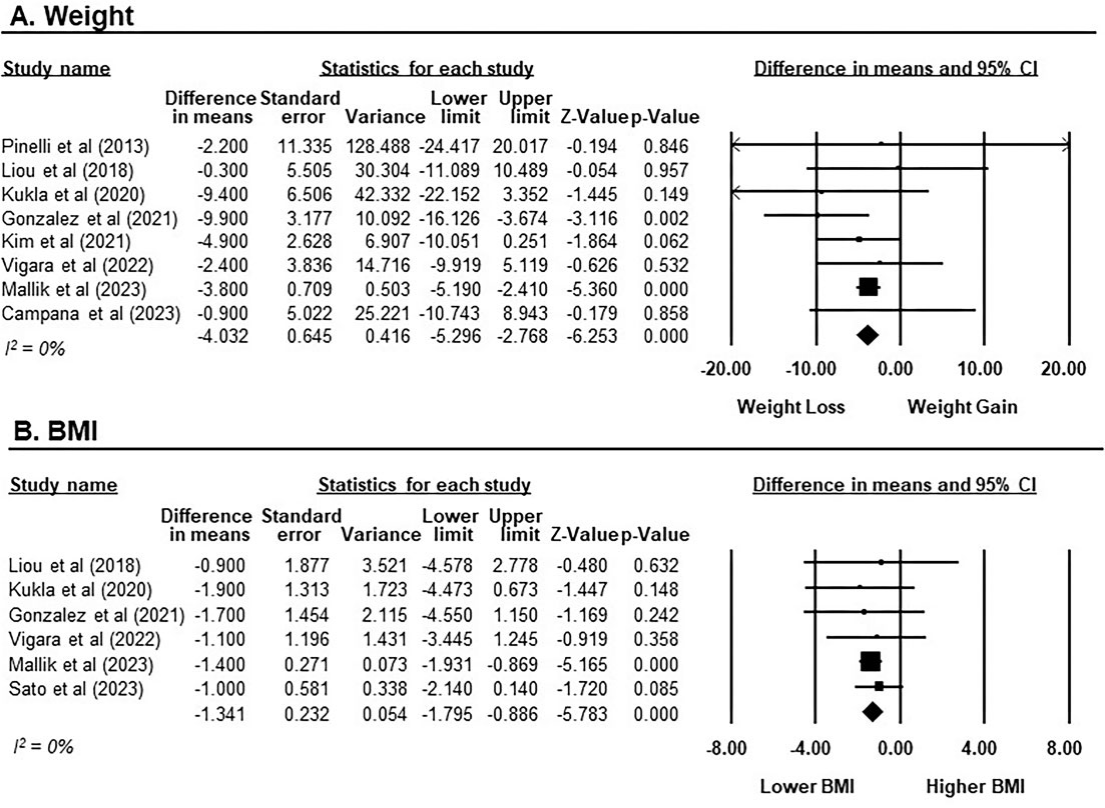
The changes in weight and BMI from baseline after GLP-1RAs treatment in KTRs. **(A)** Weight presented on a scale ranging from −20 to 20 kg. **(B)** BMI presented on a scale ranging from −8 to 8 kg/m^2^. Studies are identified by the name of the first author and the year of publication. MDs were determined using the random effects model.

Of eight studies with a total of 167 participants receiving GLP-1RAs [12, 43–49], the overall impact on weight reduction was statistically significant with an MD of −4.03 kg (95% CI −5.30 to −2.77, *P* < .001, *I*^2^ = 0%; Fig. 5A). Subgroup analysis, stratified by treatment duration, revealed that GLP-1RAs significantly reduced weight in both short-term treatment [MD −4.08 kg (95% CI −5.36 to −2.81), *P* < .001, *I*^2^ = 0%; seven studies [12, 43, 44, 46–49]] and long-term treatment [MD −4.38 kg (95% CI −7.27 to −1.50), *P* = .003, *I*^2^ = 0%; two studies [45, 46]], as shown in Supplementary Fig. S4.

In terms of BMI, the meta-analysis included six studies involving 198 participants receiving GLP-1RAs [44–48, 50]. GLP-1RAs exhibited a significant reduction in BMI compared with baseline, with an MD of −1.34 kg/m^2^ (95% CI −1.80 to −0.89, *P* < .001, *I*^2^ = 0%; Fig. 5B). Subgroup analysis stratified by treatment duration also demonstrated a significant reduction in BMI with GLP-1RAs treatment for both short-term [MD −1.30 kg/m^2^ (95% CI −1.76 to −0.83), *P* < .001, *I*^2^ = 0%; five studies [44, 46–48, 50]] and long-term use [MD −0.95 kg/m^2^ (95% CI −1.82 to −0.07), *P* = .034, *I*^2^ = 0%; three studies [45, 46, 50]], as shown in Supplementary Fig. S5.

### Efficacy of GLP-1RAs in CV and mortality outcomes

Among nine included studies with a total of 240 participants evaluated for CV and mortality outcomes, there were no reported cases of myocardial infarction, stroke or heart failure. Only one death (0.4%) was reported during the follow-up period [46]. The specific cause of death was not stated.

### Safety of GLP-1RAs in KTRs

Table 2 presents an overview of the adverse events observed in the included studies. Overall, the discontinuation rate of GLP-1RAs due to any cause was 10%. The most common reported adverse events were nausea and vomiting (17.6%), diarrhoea (7.6%) and injection site pain (5.4%). Hypoglycaemia was a rare occurrence (3.8%), reported in only three cases within one study [43]. Notably, two patients developed pancreatic diseases during GLP-1RA treatment in two separate studies: one case of pancreatitis [44] and one case of pancreatic cancer [47].

**Table 2:** Adverse events

Adverse events	Patients evaluated, *n*	Incidence, *n* (%)
Drug discontinuation due to any cause	140	14 (10)
Nausea and vomiting	119	21 (17.6)
Diarrhoea	79	6 (7.6)
Injection site pain	37	2 (5.4)
Hypoglycaemia	79	3 (3.8)
Pancreatitis	154	1 (0.6)
Pancreas cancer	154	1 (0.6)

**Figure 6:**
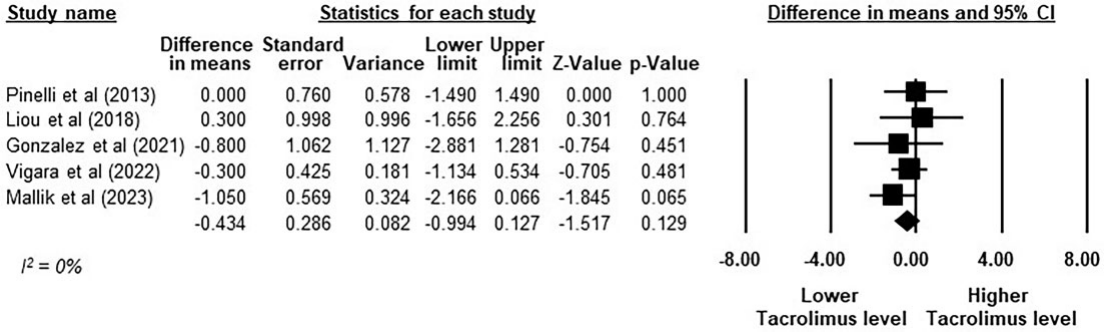
The change in tacrolimus trough level from baseline after GLP-1RAs treatment in KTRs. Studies are identified by the name of the first author and the year of publication and the outcomes are presented on a scale ranging from −8 to 8 ng/ml. MDs were determined using the random effects model.

Five studies with a total of 86 patients evaluated the impact of GLP-1RAs on immunosuppressive agents [12, 45–48]. The meta-analysis demonstrated that GLP-1RAs did not result in a significant change in tacrolimus trough levels when compared with baseline, with an MD of −0.43 ng/ml (95% CI −0.99–0.13, *P* = .129, *I*^2^ = 0%; Fig. 6). Tacrolimus dose changes following treatment with GLP-1RAs at 12 months were reported in two studies (Supplementary Table S4). Notably, only one of these studies reported a significant reduction in tacrolimus dosage in three of five patients [45]. It should be noted that no cases of graft rejection or graft dysfunction were reported in any of the nine included studies.

### Sensitivity analysis

To enhance robustness in our analysis, we conducted an additional sensitivity analysis by excluding the studies of Pinelli *et al*. [12] and Campana *et al*. [49]. The Pinelli *et al*. [12] study, despite its titled as a ‘case series’, involved systematic patient sampling based on exposures and followed them over a total study period, which is compatible with the concept of a cohort study, as described in ‘Distinguishing case series from cohort studies’ by Dekkers *et al*. [51], while the Campana *et al*. [49] study was the sole contribution sourced from a conference abstract.

Following the exclusion of Pinelli *et al*. [12], the changes of eGFR, creatinine and tacrolimus levels after GLP-1RA treatment remained comparable to the baseline with an SMD of −0.12 ml/min/1.73 m^2^ (95% CI −0.74–0.50, *P* = .710, *I*^2^ = 82%), an SMD of −0.07 mg/dl (95% CI −0.45–0.31, *P* = .724, *I*^2^ = 0%) and an MD of −0.51 ng/ml (95% CI −1.11–0.10, *P* = .102, *I*^2^ = 0%), respectively, as shown in Supplementary Fig. S6.

Upon the exclusion of Campana *et al*. [49], a statistically significant reduction in total daily insulin dose from baseline following GLP-1RA treatments persisted, yielding an MD of −7.73 units (95% CI −12.64 to −2.83, *P* = .002, *I*^2^ = 0%), as shown in Supplementary Fig. S7.

After excluding both Pinelli *et al*. [12] and Campana *et al*. [49], the impact of GLP-1RAs on weight reduction remained statistically significant, with an MD of −4.09 kg (95% CI −5.37 to −2.81, *P* < .001, *I*^2^ = 0%), as shown in Supplementary Fig. S8.

### Evaluation of publication bias

The funnel plots of standard error by SMD or MD were evaluated using Egger's regression asymmetry. These assessments revealed no indication of publication bias, as all analyses yielded *P*-values >.05, as shown in Supplementary Fig. S9.

## DISCUSSION

This is the first systematic review and meta-analysis focusing on the safety and efficacy of GLP-1RAs in KTRs. Our findings indicate that the administration of GLP-1RAs in KTRs is generally safe and without significant alterations in immunosuppressive drug levels. Moreover, these patients experience favourable outcomes in terms of proteinuria reduction, glycaemic control and weight loss, similar to the general T2DM population. Due to the limited evidence, however, we were unable to conclusively ascertain the impact of GLP-1RAs on reducing CV diseases and mortality in KTRs.

Among individuals with T2DM, a meta-analysis conducted by Sattar *et al*. [27] revealed that GLP-1RAs yield a 14% reduction in composite MACE, which encompasses CV death, myocardial infarction and stroke. Moreover, GLP-1RAs also demonstrated a reduction in all-cause mortality, hospitalization for heart failure and composite kidney outcomes while avoiding any significant increase in the risk of adverse events [27]. It is important to note, however, that all of the RCTs included in this aforementioned meta-analysis specifically excluded KTRs [23–26, 30–33, 52, 53]. Our meta-analysis was conducted to evaluate the potential use of GLP-1RAs in KTRs, a population unique in its high pill burden with potential medication interactions, increased cardiovascular risk and high risk for diabetes and its related complications.

A major safety concern for GLP-1RAs is their potential to delay gastric emptying, which could impact the absorption of immunosuppressants such as tacrolimus [12, 29]. It should be noted that our meta-analysis did not reveal any significant change in tacrolimus levels following GLP-1RA treatment. This finding was reinforced by the absence of any reported cases of graft rejection or dysfunction in any of the included studies. A possible explanation is that the metabolic pathway of GLP-1RAs primarily involves proteolytic degradation and does not interact with cytochrome P450 enzyme [47]. As a result, the likelihood of interaction with concurrent immunosuppressive drugs remains relatively low.

GLP-1RA adverse events were largely consistent with those observed in the general population [54]. Gastrointestinal (GI) side effects, particularly nausea and vomiting, were the most prevalent in our included studies, with an incidence of 17.6%. This aligns with two prior meta-analyses conducted by Bettge *et al*. [55] and Hathmacher *et al*. [56] that reported an incidence of ≈10–20% for nausea and ≈5–10% for vomiting in the non-transplant general population. Since the majority of the included studies [12, 43–45, 47] followed a protocol that favoured titration of GLP-1RAs to the highest optimal doses that patients could tolerate, this may account for the similarity in the incidence rate of GI side effects between KTRs and the general population.

The incidence of overall hypoglycaemia in KTRs receiving GLP-1RAs in our review was 3.8%, comparable to the 1–2% of severe hypoglycaemic episodes reported in a previous review of landmark RCTs [54]. Notably, hypoglycaemia risk is known to increase when GLP-1RAs are used in conjunction with sulfonylureas (SUs), insulin secretagogue medication [54]. Given that only a minority of our included patients were on SU therapy at baseline, our findings of a lower hypoglycaemia risk are consistent with the clinical context.

Regarding renal outcomes with GLP-1RAs in KTRs, we observed a significant reduction in UPCR from baseline. However, no significant effects on eGFR or serum creatinine levels were noted. This finding aligns with the prior meta-analysis by Sattar *et al*. [27], which similarly demonstrated a significant reduction in composite kidney outcomes, primarily driven by the efficacy of GLP-1RAs in reducing macroalbuminuria without significantly preventing worsening of kidney function. Conversely, it should be emphasized that our meta-analysis provides reassurance regarding the safety of initiating GLP-1RAs in KTRs since there were no significant adverse changes in eGFR or creatinine levels. This remains consistent even in light of the significant GI side effects associated with GLP-1RAs use, which may potentially lead to volume depletion and pre-renal acute kidney injury [57, 58].

Changes in glycaemic and metabolic outcomes were also evaluated in our meta-analysis. Treatment with GLP-1RAs significantly lowered HbA1c levels and reduced the total daily insulin dose in KTRs, consistent with findings in the general population [54, 59, 60]. Interestingly, a recent meta-analysis of 31 RCTs by Yeh *et al*. [60] reported a reduction in HbA1c of −0.78% (95% CI −0.97 to −0.60) and weight reduction of −4.05 kg (95% CI −5.02 to −3.09). These results closely align with our findings in KTRs, where GLP-1RAs resulted in an HbA1c reduction of −0.85% (95% CI −1.41 to −0.28) and a weight reduction of −4.03 kg (95% CI −5.30 to −2.77).

However, our meta-analysis did not demonstrate a significant effect of GLP-1RAs on BP reduction in KTRs. This is in comparison to a previous meta-analysis by Sun *et al*. [61] that demonstrated an SBP reduction of −1.84 to −4.60 mmHg with GLP-1RAs versus placebo. Due to the statistical marginal insignificance observed in our meta-analysis (*P* = .058), this disparity could be attributed to the limited number of included studies.

### Limitations

It is important to acknowledge that our systematic review has some limitations. First, among the nine studies included, only two included comparator groups. Consequently, all meta-analyses were performed by comparing outcomes with the baseline rather than the control group. Second, there was significant heterogeneity among the included studies, particularly in eGFR, UPCR and HbA1c outcomes. This may be due to follow-up duration, as one of the included studies [45] had the shortest follow-up time of 1 month. In an effort to mitigate this heterogeneity, subgroup analyses were conducted based on treatment duration. However, even stratified within these subgroups, a notable degree of heterogeneity persisted. Third, due to limited available data in included studies, subgroup analyses for the type and dosage of GLP-1RAs, relationship between GLP-1RAs and other oral hypoglycaemic drugs and types of DM (T2DM versus PTDM) were precluded. Fourth, despite an Egger's test showing no significant publication bias, the forest and funnel plots for eGFR and HbA1c changes suggest the presence of a potential publication bias and small-study effect. This finding implies that smaller studies might have disproportionately influenced the pooled SMD/MD and heterogeneity values. This recognition necessitates a cautious interpretation of our findings. Lastly, this systematic review could not assess long-term CV outcomes or death due to the short-term follow-up period of the majority of included studies. Therefore, future studies with control groups, larger sample sizes and extended follow-up periods are needed to address these limitations and validate our findings. Despite these limitations, our systematic review provides valuable insights into the safety and efficacy of GLP-1RAs among KTRs.

## CONCLUSION

While GLP-1RAs may lead to an elevated risk of GI side effects in KTRs, they demonstrate significant benefits in reducing proteinuria, improving blood glucose control and promoting weight loss while avoiding changes in tacrolimus levels.

## Supplementary Material

sfae018_Supplemental_File

## Data Availability

The data supporting this study can be found in the original publications, reports and preprints referenced in the citations.
