# Supplementary material for: Safety and efficacy of glucagon-like peptide-1 receptor agonists among kidney transplant recipients: a systematic review and meta-analysis
Source: Clin Kidney J. 2024 Feb 2;17(2):sfae018. doi: 10.1093/ckj/sfae018 (PMC10896177; doi:10.1093/ckj/sfae018)
Supplement: sfae018_Supplemental_File [file sfae018_supplemental_file.docx]

**Supplemental Material**

| **Table S1** | Search Terms |
| --- | --- |
| **Table S2** | Summary of Risk of Bias in Non-randomized Studies – of Interventions (ROBIN-I) for Included Cohort Studies |
| **Table S3** | Summary of Newcastle Ottawa Scale Scores for Included Cohort Studies |
| **Figure S1** | Subgroup Analysis of eGFR Change from Baseline in Relation to Treatment Duration |
| **Figure S2** | Subgroup Analysis of Creatinine Change from Baseline in Relation to Treatment Duration |
| **Figure S3** | Subgroup Analysis of HbA1c Change from Baseline in Relation to Treatment Duration |
| **Figure S4** | Subgroup Analysis of Weight Reduction from Baseline in Relation to Treatment Duration |
| **Figure S5** | Subgroup Analysis of BMI Reduction from Baseline in Relation to Treatment Duration |
| **Figure S6** | Sensitivity Analyses of eGFR, Creatinine, and Tacrolimus level by Excluding Pinelli et al |
| **Figure S7** | Sensitivity Analyses of Total Daily Insulin Doses by Excluding Campana et al. |
| **Figure S8** | Sensitivity Analyses of Weight Changes by Excluding Campana et al. |
| **Table S4** | Tacrolimus Dose changes |
| **Figure S9** | Funnel Plots of Standard Error |

**Table S1.** Search Terms

| 1 | GLP-1 receptor agonist*.mp. |
| --- | --- |
| 2 | GLP-1 RA.mp. |
| 3 | Glucogon-like peptide-1 receptor agonist*.mp. |
| 4 | liraglutide.mp. |
| 5 | semaglutide.mp. |
| 6 | dulaglutide.mp. |
| 7 | lixisenatide.mp. |
| 8 | exenatide.mp. |
| 9 | albiglutide.mp. |
| 10 | efpeglenatide.mp. |
| 11 | 1 or 2 or 3 or 4 or 5 or 6 or 7 or 8 or 9 or 10 |
| 12 | kidney transplant*.mp. |
| 13 | renal transplant*.mp. |
| 14 | 12 or 13 |
| 15 | 11 and 14 |
| 16 | limit 15 to human |

**Table S2.** Summary of Risk of Bias in Non-randomized Studies – of Interventions (ROBIN-I) for the Included Cohort Studies

| **Bias due to** | **Pinelli et al. (2013)** | **Liou et al. (2018)** | **Kukla et al. (2020)** | **Gonzalez et al. (2021)** | **Kim et al. (2021)** | **Vigara et al. (2022)** | **Mallik et al (2023)** | **Sato et al. (2023)** | **Campana et al. (2023)** |
| --- | --- | --- | --- | --- | --- | --- | --- | --- | --- |
| Confounding | Low | Low | Low | Low | Low | Low | Low | Low | Low |
| Selection | Low | Low | Low | Low | Low | Low | Low | Low | Low |
| Classification of interventions | Moderate | Moderate | Moderate | Moderate | Moderate | Moderate | Moderate | Moderate | Moderate |
| Deviations from intended interventions | Low | Low | Low | Low | Low | Moderate | Low | Low | Low |
| Missing data | Low | Low | Low | Low | Low | Moderate | Moderate | Low | Low |
| Measurement outcome | Moderate | Moderate | Moderate | Moderate | Moderate | Moderate | Moderate | Moderate | Moderate |
| Selection of reported result | Low | Low | Low | Low | Low | Low | Low | Low | Low |
| **Overall** | Moderate | Moderate | Moderate | Moderate | Moderate | Moderate | Moderate | Moderate | Moderate |

**Table S3.** Summary of Newcastle Ottawa Scale Scores for the Included Cohort Studies

| **Author (year)** | **Selection** | **Comparability** | **Outcome** | **Scores** |
| --- | --- | --- | --- | --- |
| Pinelli et al. (2013) | *0** | ** | *0* | 7 |
| Liou et al. (2018) | *0** | ** | *** | 8 |
| Kukla et al. (2020) | *0** | ** | *** | 8 |
| Gonzalez et al. (2021) | *0** | 0* | *** | 7 |
| Kim et al. (2021) | *0** | ** | *0* | 7 |
| Vigara et al. (2022) | *0** | 0* | **0 | 6 |
| Mallik et al (2023) | *0** | ** | **0 | 7 |
| Sato et al. (2023) | **** | ** | *** | 9 |
| Campana et al. (2023) | **** | ** | *** | 9 |
|  |  |  |  |  |


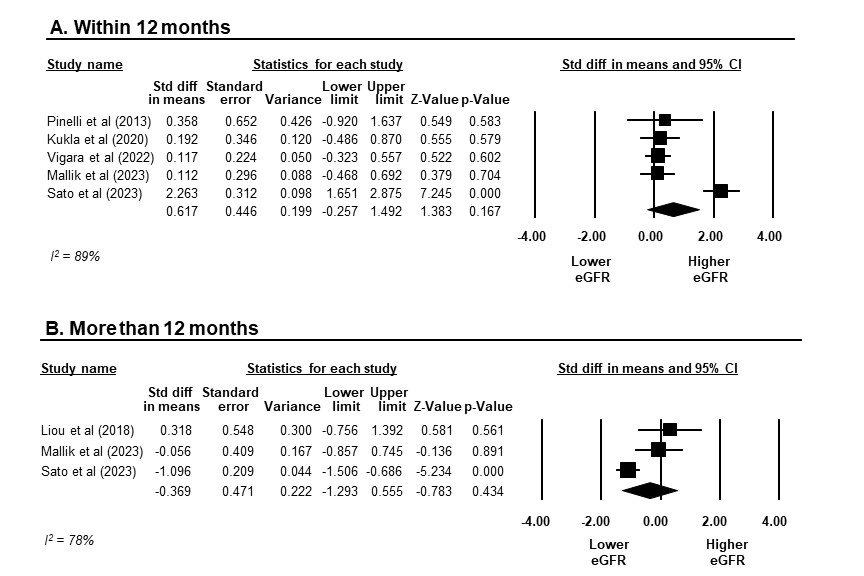


**Figure S1.** Subgroup Analysis of eGFR Change from Baseline in Relation to Treatment Duration. (A) Within 12 months of GLP-1RAs treatment, (B) More than 12 months of GLP-1RAs treatment. Studies are identified by the name of the first author and the year of publication. Standard mean differences were performed using the random-effects model and presented on a scale ranging from -4 to 4 ml/min/1.73m^2^. Abbreviations: CI, confident interval; eGFR, estimated glomerular filtration rate, GLP-1RAs, glucagon-like peptide-1 receptor agonists.


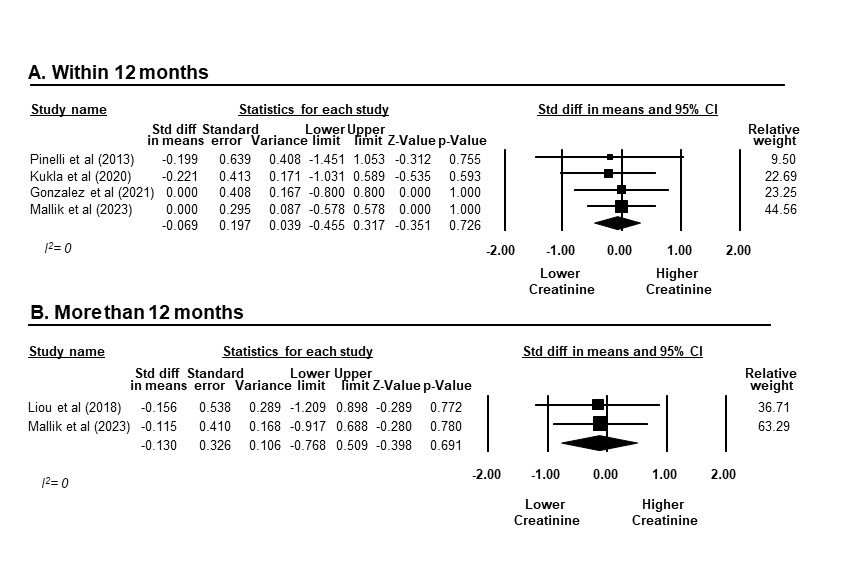


**Figure S2.** Subgroup Analysis of Creatinine Change from Baseline in Relation to Treatment Duration. (A) Within 12 months of GLP-1RAs treatment, (B) More than 12 months of GLP-1RAs treatment. Studies are identified by the name of the first author and the year of publication. Standard mean differences were performed using the random-effects model and presented on a scale ranging from -2 to 2 mg/dL. Abbreviations: CI, confident interval; GLP-1RAs, glucagon-like peptide-1 receptor agonists.


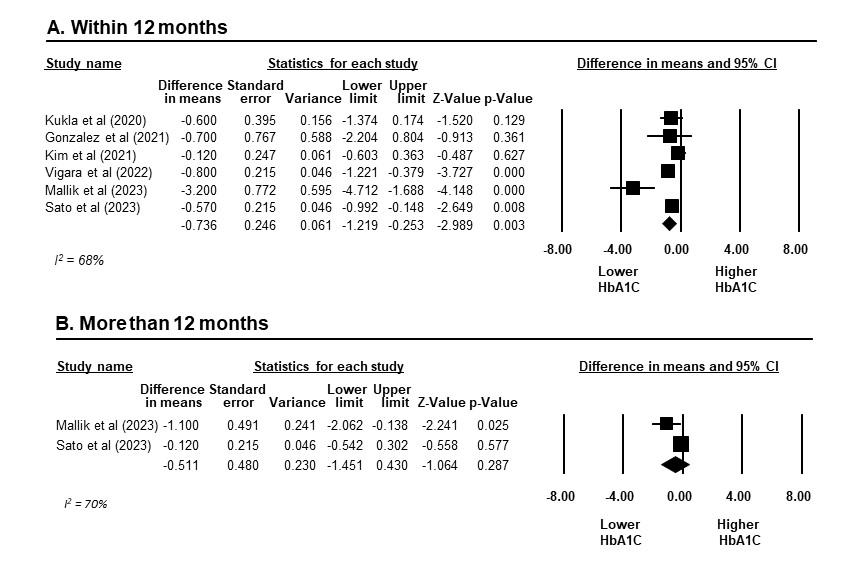


**Figure S3.** Subgroup Analysis of HbA1c Change from Baseline in Relation to Treatment Duration. (A) Within 12 months of GLP-1RAs treatment, (B) More than 12 months of GLP-1RAs treatment. Studies are identified by the name of the first author and the year of publication. Mean differences were performed using the random-effects model and presented on a scale ranging from -8 to 8%. Abbreviations: CI, confident interval; GLP-1RAs, glucagon-like peptide-1 receptor agonists.


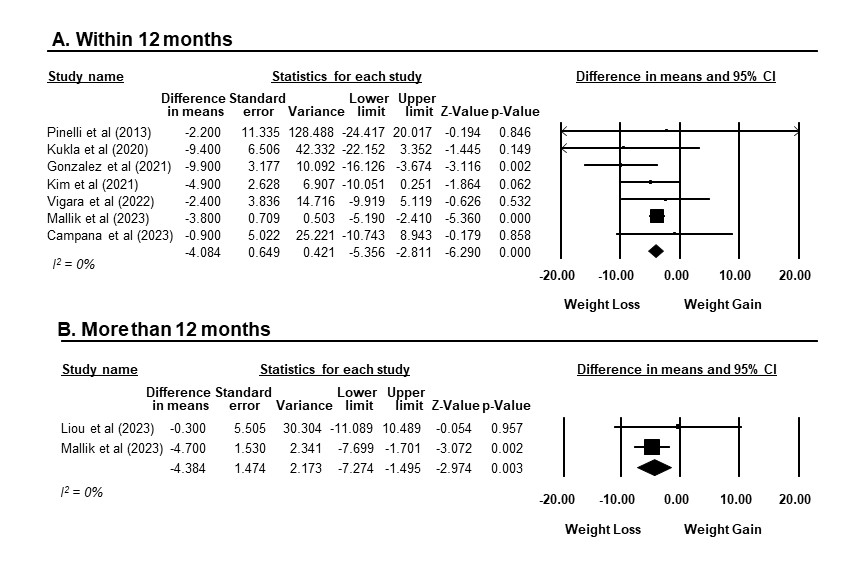


**Figure S4.** Subgroup Analysis of Weight Reduction from Baseline in Relation to Treatment Duration. (A) Within 12 months of GLP-1RAs treatment, (B) More than 12 months of GLP-1RAs treatment. Studies are identified by the name of the first author and the year of publication. Mean differences were performed using the random-effects model and presented on a scale ranging from -20 to 20 kg. Abbreviations: CI, confident interval; GLP-1RAs, glucagon-like peptide-1 receptor agonists.


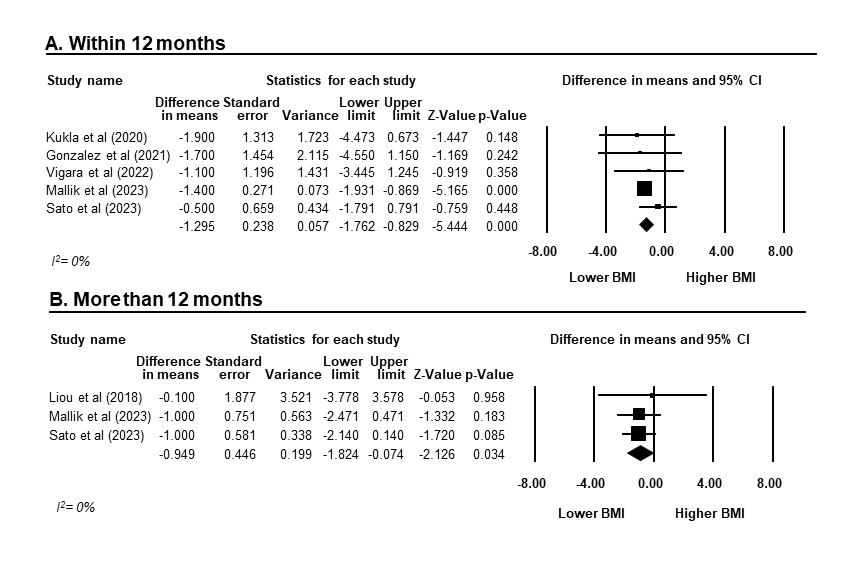


**Figure S5.** Subgroup Analysis of BMI Reduction from Baseline in Relation to Treatment Duration. (A) Within 12 months of GLP-1RAs treatment, (B) More than 12 months of GLP-1RAs treatment. Studies are identified by the name of the first author and the year of publication. Mean differences were performed using the random-effects model and presented on a scale ranging from -20 to 20 kg. Abbreviations: BMI, body mass index; CI, confident interval; GLP-1RAs, glucagon-like peptide-1 receptor agonists.

**Table S4.** Tacrolimus Dose Changes

| **Author (year)** | **N** | **Time assessment (months)** | **Total Tacrolimus Doses (mg/day)** | |
| --- | --- | --- | --- | --- |
|  |  |  | **Baseline** | **After GLP-1RAs** |
| **Liou et al (2018)** | 5 | 12 | 5.9 ± 1.7 | 5.5 ± 1.9 |
| **Vigara et al (2022)** | 26 | 12 | 3 ± 2.4 | 2.5 ± 1.4 |


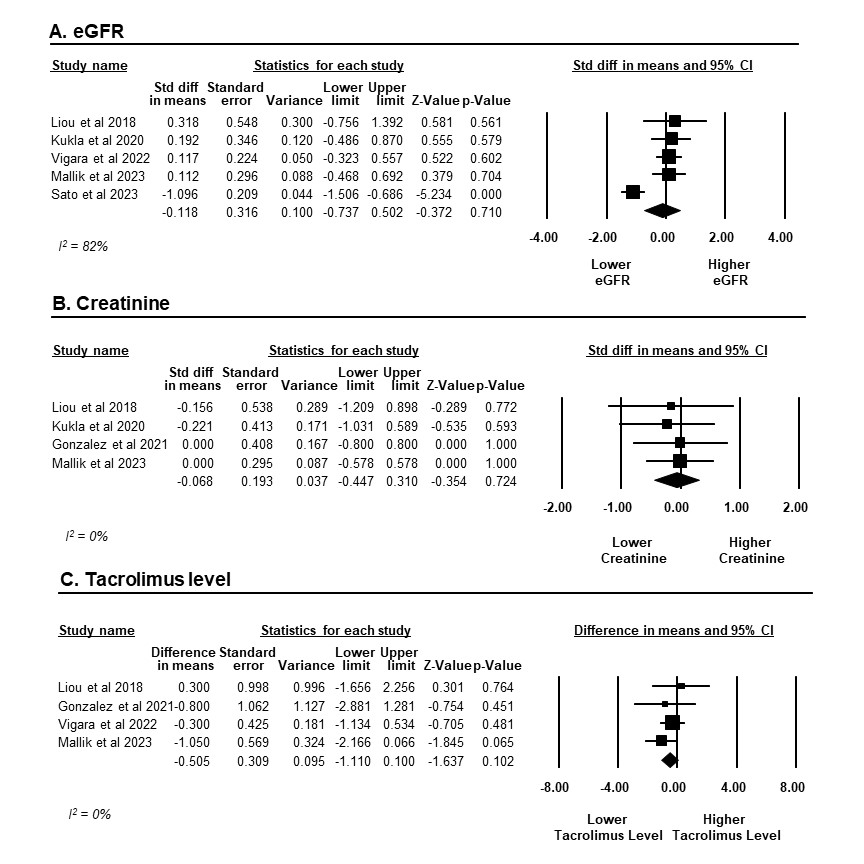


**Figure S6.** Sensitivity Analyses by Excluding Pinelli et al (A) eGFR (B) Creatinine (C) Tacrolimus level. Studies are identified by the name of the first author and the year of publication. Abbreviations: CI, confident interval; eGFR, estimated glomerular filtration rate.


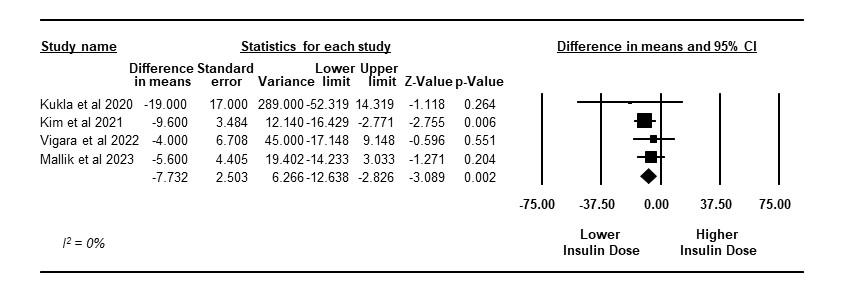


**Figure S7.** Sensitivity Analyses of Total Daily Insulin Doses by Excluding Campana et al. Studies are identified by the name of the first author and the year of publication. Abbreviations: CI, confident interval.


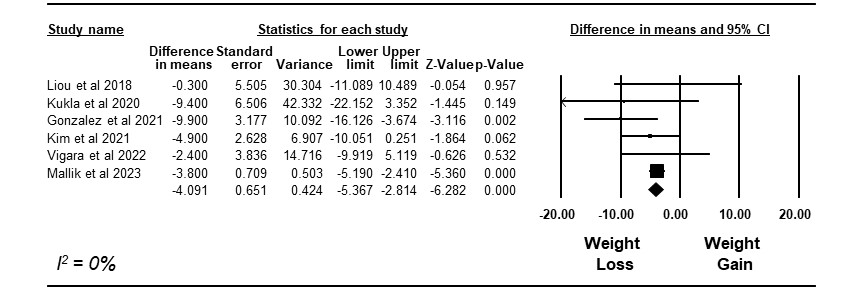


**Figure S8.** Sensitivity Analyses of Weight Changes by Excluding Pinelli et al and Campana et al. Studies are identified by the name of the first author and the year of publication. Abbreviations: CI, confident interval.


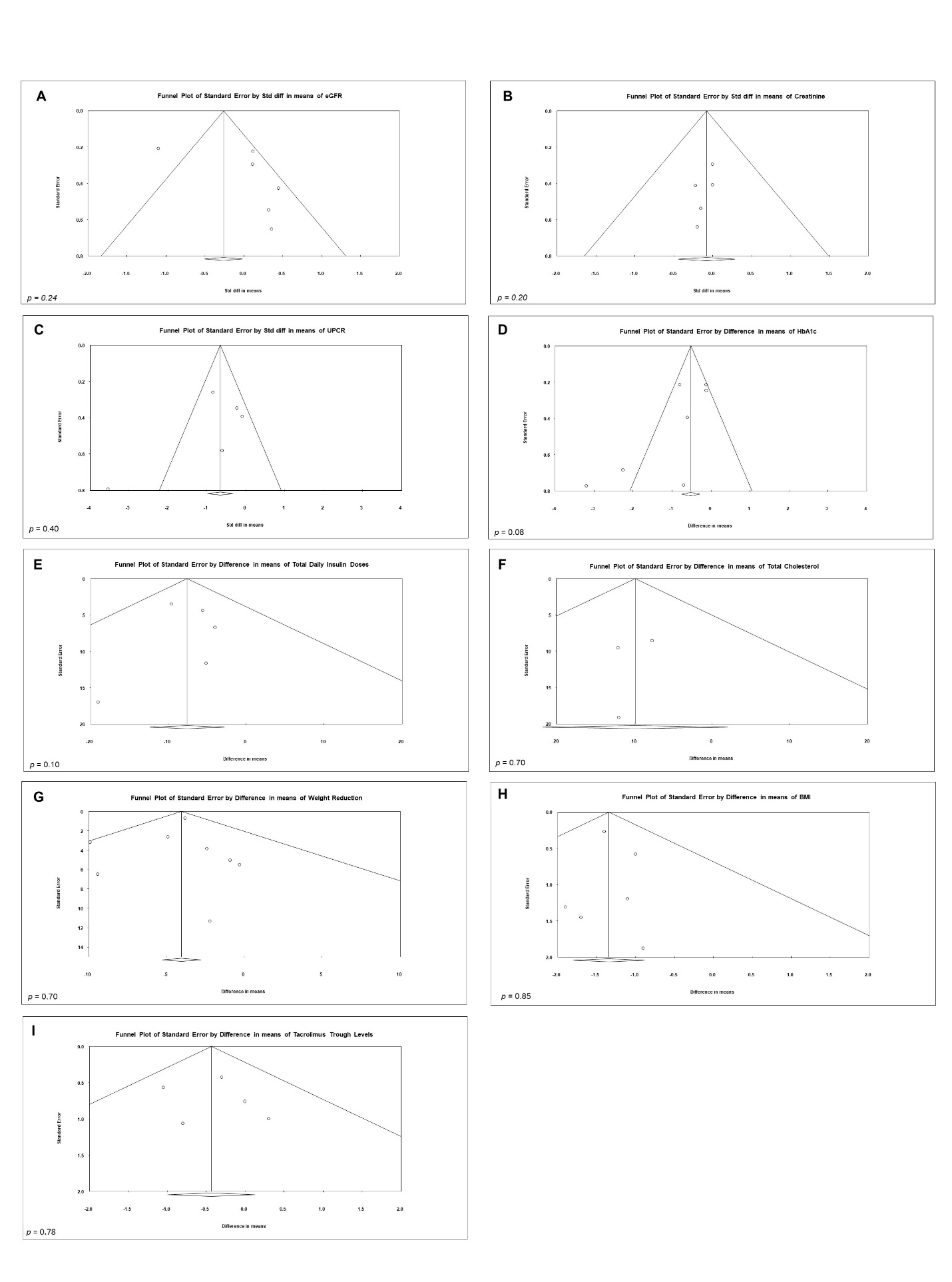


**Figure S9.** Funnel Plots of Standard Error (A) eGFR, (B) Creatinine, (C) UPCR, (D) HbA1c, (E) Total Daily Insulin Doses, (F) Total Cholesterol, (G) Weight Reduction, (H) BMI, (I) Tacrolimus Trough Level. Studies are identified by the name of the first author and the year of publication. Mean differences were performed using the random-effects model and presented on a scale ranging from -20 to 20 kg. Abbreviations: BMI, body mass index; CI, confident interval; eGFR, estimated glomerular filtration rate; GLP-1RAs, glucagon-like peptide-1 receptor agonists; UPCR, urine-protein creatinine ratio.
